# P-105. Safety and Immunogenicity of BNT163, a Trivalent mRNA HSV Vaccine Candidate for Genital Herpes

**DOI:** 10.1093/ofid/ofaf695.334

**Published:** 2026-01-11

**Authors:** Akira A Shishido, Pablo Tebas, Claudia S Crowell, Sushma Kommineni, Gosford Sawyerr, Bonnie S Bielefeld, Wen Ding, Tania Fritsch, Raquel Furtado, Robbert G van der Most, Orkun Ozhelvaci, Sita Awasthi, Gary H Cohen, Harvey M Friedman, Federico Mensa

**Affiliations:** BioNTech US Inc, Cambridge, Massachusetts; University of Pennsylvania, Philadelphia, Pennsylvania; BioNTech SE, Mainz, Hamburg, Germany; BioNTech US Inc, Cambridge, Massachusetts; BioNTech US Inc, Cambridge, Massachusetts; Fortrea, Durham, North Carolina; BioNTech US Inc, Cambridge, Massachusetts; BioNTech SE, Mainz, Hamburg, Germany; BioNTech US Inc, Cambridge, Massachusetts; BioNTech SE, Mainz, Hamburg, Germany; BioNTech SE, Mainz, Hamburg, Germany; Perelman School of Medicine, University of Pennsylvania, Philadelphia, Pennsylvania; University of Pennsylvania, Philadelphia, Pennsylvania; Perelman School of Medicine, University of Pennsylvania, Philadelphia, Pennsylvania; BioNTech, Mainz, Rheinland-Pfalz, Germany

## Abstract

**Background:**

Herpes simplex virus type 2 (HSV-2) affects ∼12% of individuals aged 14–49 years in the US. HSV-2-related genital herpes is a life-long and stigmatizing infection, often marked by recurrent episodes that can cause painful lesions and increase transmission of other sexually transmitted infections. BNT163 is an investigational lipid nanoparticle-encapsulated trivalent mRNA vaccine, encoding HSV-2 glycoproteins gC2, gD2, and gE2, which demonstrated immunogenicity and prevention of genital lesions in preclinical HSV-2 models.

**Table 1.**
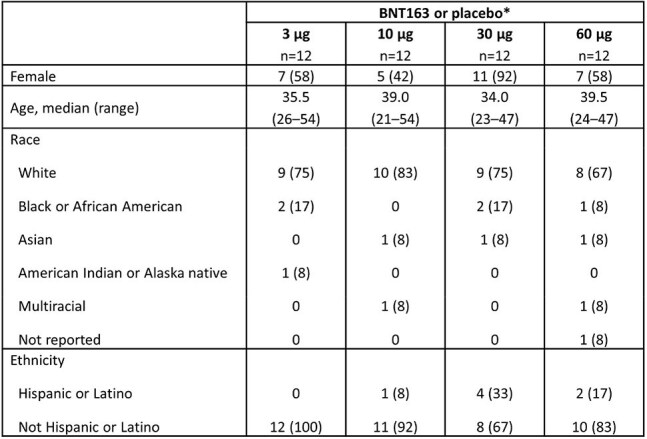
Baseline demographics Data are n (%) unless otherwise stated. *In each group, 10 participants were randomized to BNT163 and two to placebo.

**Figure 1.**
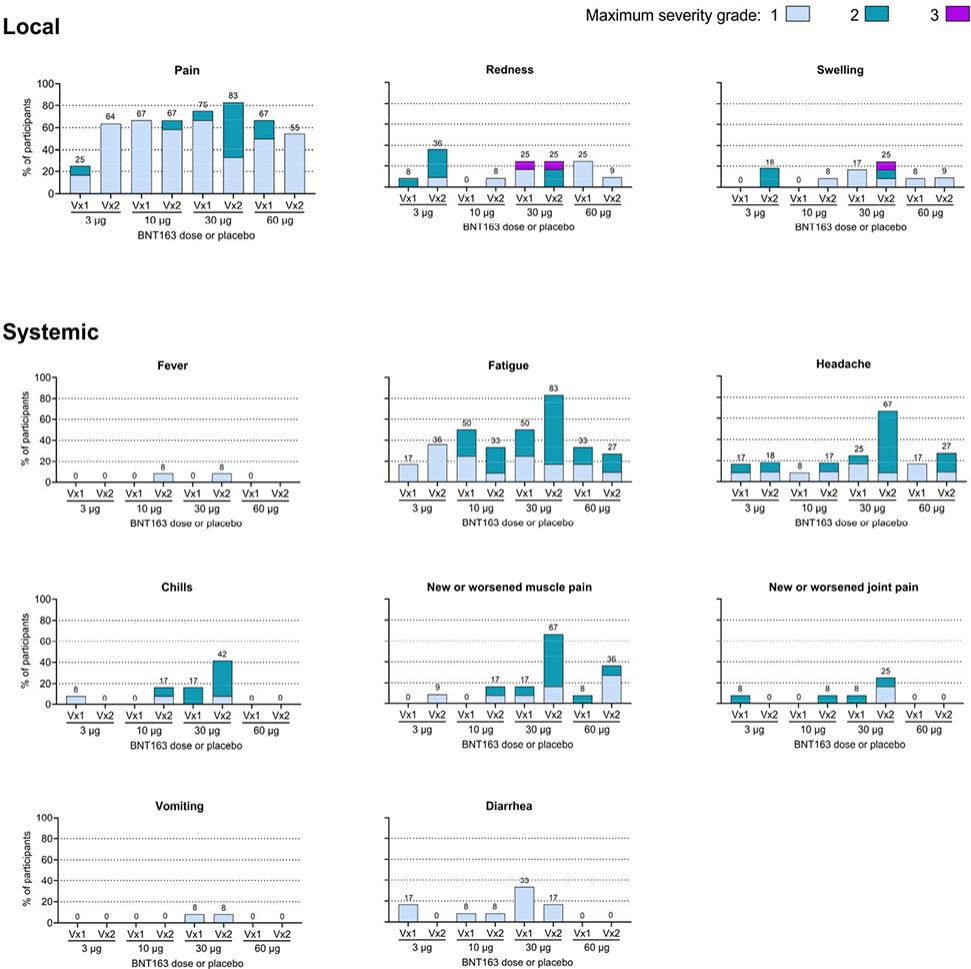
Local and systemic reactogenicity events occurring within 7 days of Vaccinations 1 and 2 Vx, vaccination.

In total, 12 participants in each group received Vaccination 1 of BNT163 or placebo, and 11, 12, 12 and 11 received Vaccination 2 or placebo in the 3 µg, 10 µg, 30 µg and 60 µg groups, respectively. Reactogenicity events were participant-reported in electronic diaries for 7 days after each vaccination. Numbers above each bar denote the percentage of participants in that cohort who experienced the reaction with any severity. Local. Pain at the injection site was graded as 1 (mild, does not interfere with activity), 2 (moderate, interferes with activity), 3 (severe, prevents daily activity) or 4 (potentially life-threatening, emergency room visit or hospitalization). Redness and swelling were graded as 1 (mild, 2.5–5.0 cm in diameter), 2 (moderate >5.0–10.0 cm), 3 (severe, >10.0 cm), or 4 (necrosis or exfoliative dermatitis for redness and necrosis for swelling). Systemic. Chills, fatigue, headache, muscle pain and joint pain were graded as 1 (mild, does not interfere with activity), 2 (moderate, some interference with activity), 3 (severe, prevents daily routine activity) or 4 (potentially life-threatening, emergency room visit or hospitalization). Diarrhea was graded as 1 (mild, 2–3 loose stools in 24 hours), 2 (moderate, 4–5 loose stools in 24 hours), 3 (severe, ≥6 loose stools in 24 hours) or 4 (potentially life-threatening, emergency room visit or hospitalization). Fever was graded as 1 (mild, 38.0°C–38.4°C), 2 (moderate, 35.8°C–38.9°C), 3 (severe, 39.0°C–40.0°C), or 4 (potentially life-threatening, >40.0°C). Vomiting was graded as 1 (mild, 1–2 times in 24 hours), 2 (moderate, >2 times in 24 hours), 3 (severe, requires intravenous hydration), or 4 (potentially life-threatening, emergency room visit or hospitalization).

**Methods:**

In this ongoing first-in-human dose-escalation Phase 1 trial (NCT05432583), healthy participants (18–55 years) without history of symptomatic genital herpes were assigned to receive intramuscular injections of 3, 10, 30, or 60 µg BNT163, or placebo, on Days 1, 56 and 112. In each dose group, 12 participants were randomized to BNT163 (n=10) or placebo (n=2). Dose escalation proceeded based on predefined safety and tolerability criteria, as reviewed and approved by an internal review committee. The primary endpoint is safety, with secondary and exploratory endpoints assessing immunogenicity. Blinded data are reported through Day 28 post-Vaccination (Vx) 2.

**Figure 2.**
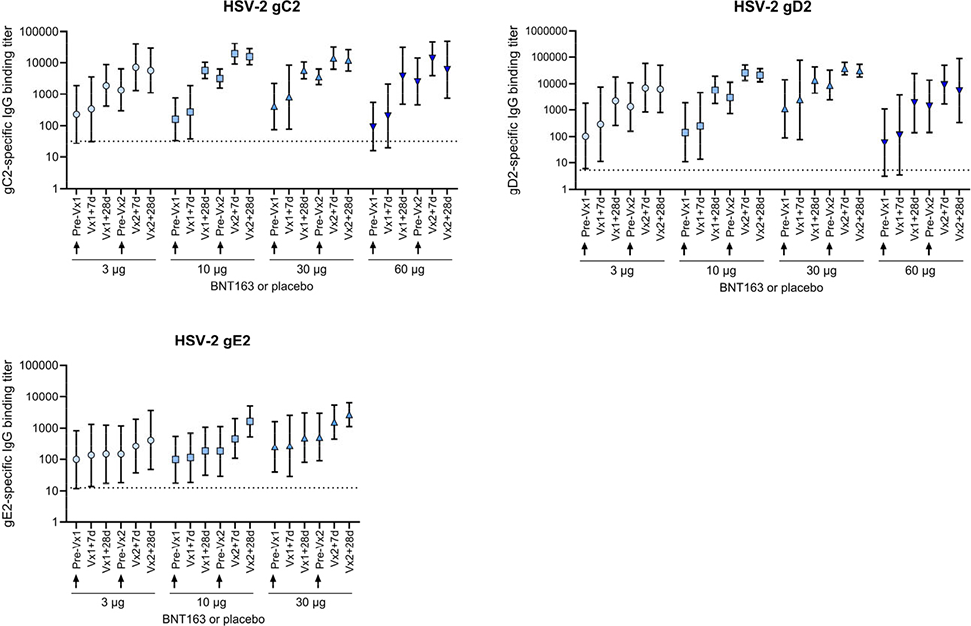
Geometric mean and 95% confidence intervals of antigen-specific antibody binding titers in evaluable immunogenicity sets through 28 days post-Vaccination 2 d, days; IgG, immunoglobulin G; Vx, vaccination.

BNT163 or placebo was administered on Days 1 and 56 (arrows indicate day of administration). Serum samples were obtained pre-Vx1, 7 and 28 days post-Vx1, pre-Vx2, and 7 and 28 days post-Vx2.

Titers of binding IgG are shown for HSV-2 glycoproteins gC2, gD2, and gE2. Dashed lines indicate LLOQ for each antigen: 32.6, 5.3, and 12.6 AU/mL for gC2, gD2, and gE2, respectively. gE2-specific titers for the 60 µg group were not available at the time of analysis but will be presented at the meeting.

**Results:**

Forty-eight participants enrolled in this part of the trial and received at least one administration of BNT163 or placebo. Baseline demographics were balanced across groups, except for a higher proportion of female participants in the 30 µg group (Table 1). Local and systemic reactogenicity events were mostly mild-to-moderate across dose groups (Figure 1) and no safety pausing or stopping rules were met. With data blinded at time of submission, the geometric mean binding antibody titers to all encoded antigens for available cohorts showed an increasing trend following Vx 1 and Vx 2 (Figure 2). HSV-2 neutralization titers were induced across dose groups available for analysis at the time of submission. Unblinded safety, antibody and cell-mediated immune response data post-Vx 3 will be presented for each dose group and combined placebo group.

**Conclusion:**

BNT163 is well-tolerated with an acceptable safety profile and induces binding antibody and neutralizing titers to HSV-2 antigens, supporting its continued clinical development.

**Disclosures:**

Akira A. Shishido, MD, BioNTech US Inc: Employee Pablo Tebas, MD, BioNTech SE: Grant/Research Support|Hoopkipa: Grant/Research Support|Inovio: Grant/Research Support|Merck: Advisor/Consultant|Merck: Grant/Research Support|Shionogi: Advisor/Consultant|Shionogi: Grant/Research Support|ViiV Healthcare: Advisor/Consultant|ViiV Healthcare: Grant/Research Support Claudia S. Crowell, MD, MPH, BioNTech SE: Employee|BioNTech SE: Stocks/Bonds (Public Company) Sushma Kommineni, PhD, BioNTech: Employee|BioNTech: Stocks/Bonds (Public Company) Gosford Sawyerr, MA, BioNTech US Inc: Employee Wen Ding, MS, BioNTech: Employee|BioNTech: Stocks/Bonds (Public Company) Tania Fritsch, MSc, BioNTech SE: Employee Raquel Furtado, PhD, BioNTech: BioNTech SE|BioNTech: Employee|BioNTech: Stocks/Bonds (Public Company) Robbert G. van der Most, PhD, BioAster: Advisor/Consultant|BioNTech SE: Advisor/Consultant|BioNTech SE: Stocks/Bonds (Public Company)|Gates Foundation: Advisor/Consultant|P-95: Advisor/Consultant|Sanofi: Advisor/Consultant|WHO: Advisor/Consultant Orkun Ozhelvaci, BSc, BioNTech SE: Employee|BioNTech SE: Stocks/Bonds (Public Company) Sita Awasthi, PhD, BioNTech SE: Grant/Research Support|BioNTech SE: License from Penn to BioNTech. H Friedman, G. Cohen, S Awasthi are inventors on the patent Penn licensed to BioNTech|BioNTech SE: Partial support was provided for attending and presenting at Biologics conference in Goa India 2025 January for registration and travel|Merck: Royalties from Merck for a protein-based subunit vaccine|NIH: Grant/Research Support Gary H. Cohen, PhD, BioNTech US: Funding provided for abstract submission Harvey M. Friedman, MD, BioNTech: License from Penn to BioNTech. H Friedman, G. Cohen, S Awasthi are inventors on the patent Penn licensed to BioNTech|BioNTech: Support with abstract submission|Merck: Royalties from Merck for a protein-based subunit vaccine|NIH: Grant/Research Support|NIH: Travel support for meetings|University of Pennsylvania: Awarded Patents|Wolters Kluwer: Honoraria Federico Mensa, MD, BioNTech US: Honoraria|BioNTech US: Employee|BioNTech US: Stocks/Bonds (Public Company)

